# Should I stay or should I go? Three-year-olds’ reactions to appropriate motives to interrupt a joint activity

**DOI:** 10.1371/journal.pone.0288401

**Published:** 2023-07-13

**Authors:** Francesca Bonalumi, Barbora Siposova, Wayne Christensen, John Michael

**Affiliations:** 1 Department of Cognitive Science, Central European University, Vienna, Austria; 2 Department of Philosophy, University of Warwick, Coventry, United Kingdom; 3 Department of Psychology and Life Science, Charles University, Prague, Czech Republic; 4 Department of Psychology, University of Warwick, Coventry, United Kingdom; 5 Department of Philosophy, University of Milan, Milan, Italy; University of Guelph, CANADA

## Abstract

Understanding when it is acceptable to interrupt a joint activity is an important part of understanding what cooperation entails. Philosophical analyses have suggested that we should release our partner from a joint activity anytime the activity conflicts with fulfilling a moral obligation. To probe young children’s understanding of this aspect, we investigated whether 3-year-old children (*N* = 60) are sensitive to the legitimacy of motives (selfish condition vs. moral condition) leading agents to intentionally interrupt their joint activity. We measured whether children protested or released their partner by scoring their reactions. Our results indicate that children did not manifest different reactions when the motive behind their partner leaving was moral than when the motive was selfish. However, our data showed a stable pattern: regardless of the partner’s motives, some 3-year-olds take initiatives to release their partners from joint activity, suggesting that measuring release is a valuable tool for investigating joint action.

## Introduction

Most of human social life is based on coordinating with social partners to achieve greater goals than we could achieve alone, but also to build reciprocal trust and cultivate relationships. Any social activity requires us to predict and adjust to our partners’ behaviors, but such predictions are difficult when partners face potential conflicting motivations. Understanding when to expect partners to complete a joint activity are thus key skills for navigating the social world [1–6].

However, understanding when it is appropriate to update one’s own expectations and release partners from acting as one expects is also an important part of understanding what collaborations entail. In fact, every time a collaboration is in place people have priors about its scope and priority. When is it the case that someone is expected *to be released from* the collaboration? Philosophical analyses have suggested that we should release our partner anytime this conflicts with fulfilling a moral obligation [7]. Imagine, for example, Sarah went hiking on a Sunday afternoon. After some time, she met Melissa and they started to hike together. If Sarah suddenly changes her mind and leaves to join other friends in a bar downtown without any acknowledgment, Melissa is likely to be annoyed and to expect an explanation or an apology from Sarah. However, if Sarah is a medical doctor and leaves to assist a man who passes out, it would be strange for Melissa to expect that Sarah follow through on their hiking activity. In both cases, Sarah intentionally interrupts her hike with Melissa; however, she has different motives in the two cases. In the first case, their joint activity conflicts with a selfish motive to have more fun with other friends. In the second case, there is a conflict with Sarah’s moral obligation towards her patient—this obligation should outweigh any other consideration [as argued by e.g., 7].

To our knowledge, this idea has never been tested empirically, either in adults or children [for a recent exception, see 8]. We thus empirically investigated whether participants release their partner from their joint activity when this conflicts with a moral duty. We designed a novel cooperative game that required two partners to coordinate their actions to obtain rewards. In the selfish motive condition, the partner left the game to play another game; in the moral motive condition, the partner left to help another individual. We predicted that participants would release their partner from the collaboration more frequently in the moral motive condition compared to the selfish motive condition.

Previous research has shown that children as young as 3 years of age start to form joint goals when engaged in joint activities [9–11]. Furthermore, when children are in collaborative settings, preschoolers have been found to be more motivated to invest effort and resist temptations, as well as to persist in a joint activity. For example, Koomen and colleagues showed how preschoolers delay gratifications in a joint marshmallow test [12]. Coordinated joint activities can induce motivation to reach joint goals [11], and such motivation can be induced even when partners are not present—as in cases in which children are merely told to be engaged in a joint task [13].

While these behaviors are indirectly suggesting that children might perceive normative obligations out of their collaborative joint activities, one typical interpretation of these behaviors is that, during these activities, children inferred that commitments between themselves and their collaborative partner are in place. Commitments are particularly useful tools, because they reduce uncertainty about others’ behavior by stabilizing motivations and helping us to rely on one another [14]. Commitments also ground normative obligations, which entitle individuals to reproach partners who interrupt joint activities [1, 15, 16]. Along these lines, three-year-olds have been found to distinguish between situations in which their partner interrupts a joint activity and breaks their commitment intentionally (for selfish motives) and situations in which they do so unintentionally (by accident or due to inability) [17]. Recent research investigated whether children distinguish between situations in which they themselves, partners and a third agent had either selfish or moral motives to break an explicit commitment (i.e., a promise) [8, 18]. Both 3- and 5-year-olds equally committed to keep their own promise no matter the motive for breaking it; however, when asked to choose the ending of a story involving a third-parties social interaction, older children chose the promise-breaking ending more when there were moral motives in comparison to selfish motives, while younger children showed the opposite attitude [18]—although it is difficult to distinguish whether such choice reflected children’s prediction about the agent behavior or a weighting of their motives. On the other hand, when a partner broke an explicit promise, both 3- and 5-year-olds judged the partner less negatively when the promise violation was justified with a good (prosocial) motive [8]. In this study, children were given the option to tattle on the promise violator, to evaluate the wrongness of their violation, as well as to report whether they liked the violator and whether they would choose them as collaborators in the future. The motives behind the promise-breaking influence children’s judgments about its wrongness, but did not have any impact on the three other measures.

In contrast to Kanngiesser and colleagues’ [18] studies, which focused on children’s evaluation of third-party behavior and motivation to keep *their own* promise [8], we tested children’s reactions *when their partner* interrupted their joint activity. In the current study, our main aim was to investigate whether children are sensitive to the motives leading their own partners to interrupt their joint activity, i.e., when children are the ones suffering the consequences of the interruption. This second-person approach was used also by Li and colleagues [8], but in contrast to them, we adopted a protest paradigm [see 17, 19, 20], instead of measuring their explicit evaluation of the situation. In addition, and critically in contrast to both Kanngiesser and colleagues [18] and Li and colleagues [8], in this study we did not orchestrate a verbal agreement between the participant and their partner; participants were instead put in a repeated joint activity context, in which they were interdependent and coordinated with their partners [21, 22]. Even though commitment cannot be measured directly, it has been argued that repeated joint activities and interdependence are factors that give rise to a sense of commitment [3, see also 21], and there are empirical findings suggesting that people infer commitments in such situations [12, 23–26]. We predicted that children would be more likely to release their partner from their joint activity when this conflicts with a moral duty, compared to a situation in which this conflicts with a selfish motive.

Previous literature has focused on detecting children’s signs of protest or explicit judgments (see Table 1) overlooking signs of release that would indicate a finer appreciation of the scope and priority of the established joint activity and possible implicit commitment. In the present study, we designed *a release measure*—which not only captures protest (denied release), but also children’s tendency to actively free their partner from their joint activity (release).

**Table 1:** Review of studies investigating children’s understanding of the scope of cooperative activities.

Study	Role in the potential commitment	Factors that might induce commitment	Measure	Finding
Gräfenhain et al. 2009 Study 1	2^nd^ person	Explicit: promise	Expecting the experimenter to play	3- but not 2-year-olds expect the experimenter to re-engage when a joint commitment was in place.
Gräfenhain et al. 2009 Study 2	1^st^ person	Explicit: promise	Leave-taking behavior	3- and 4-year-olds acknowledge their commitment breaking when a joint commitment was previously in place.
Hamann et al. 2012	2^nd^ person	Implicit:	Collaborative helping	3- but not 2-year-olds provide collaborative help to partners.
Butler and Walton, 2013	1^st^ person	Implicit: we-framing	Persistence in a task	4- and 5-year-olds persisted longer in a task when they believed that they were doing the task together with their partner.
Kachel et al., 2018	2^nd^ person	Explicit	Protest, tattling, and teaching	3-year-olds distinguished between intentional and non-intentional commitment breakings.
Siposova et al., 2018	2^nd^ person	Implicit: nonverbal communicative look	Protest	6-7-year-olds understood a communicative looks as a commitment and protested to commitment breakings.
Kachel et al., 2019	2^nd^ person	Explicit	Protest	3-year-olds react differently to excused and non-excused commitment breakings.
Kachel & Tomasello, 2019	1^st^ person	Explicit: promise; Implicit: interdependent outcomes	Bribe accept	3-to-5-year-olds resist bribes even when commitment is implicit, though to a lesser degree.
Koomen et al., 2020	1^st^ person	Implicit: interdependent outcomes	Delay gratification	5-to 6-year-olds were more likely to delay gratification when their outcomes were interdependent.
Kanngiesser et al., 2021 Study 1	1^st^ person	Explicit: promise	Keep promise	3- and 5-year-olds keep their promises no matter the motive to break it.
Kanngiesser et al., 2021 Study 2	3^rd^ person	Explicit: promise	Ending choice	5- but not 3-year-olds prefer situations when agents break their promise for moral motive.
Li et al., 2023	2^nd^ person	Explicit: promise	Explicit judgments: tattling, wrongness, liking, invitation	3- and 5-year-olds judge agents less negatively when they break their promises because of a moral motive, but only 5-year-olds can justify their choices.
Current Study	2^nd^ person	Implicit: Interdependent outcomes	Protest-release	Do 3-year-olds react differently to moral and selfish commitment breakings?

Our secondary aim was to investigate to what extent children’s sophistication to understand the scope of commitments is related to their cognitive skills, namely understanding others’ mental states and justificatory abilities. It has been suggested that children’s theory of mind skills are related to their appreciation of commitments–specifically, their ability to understand agents’ motives when undertaking a commitment [27]. Recognizing others’ beliefs and desires is an important component of the development of moral judgment, as it is only later in childhood that children begin to explicitly punish agents for having malevolent intentions rather than for causing a negative outcome [28]; on the other hand, it has been shown that much younger children can both recognize agents’ malevolent intentions and socially evaluate them accordingly [29, 30]. We thus reasoned that those participants who better recognized others’ mental states, and more specifically others’ desires, will be better able to assess the appropriateness of the partner’s motive. Therefore, we predicted that mentalizing skills would predict an increased tendency to release the partner in the moral condition only. Additionally, violations of moral expectations are often followed by persuasive attempts to justify one’s actions [31]; previous research has shown 3-year-olds’ preference for non-circular arguments, suggestive of an appreciation of what counts as good justification [see e.g., 32–34]. We reasoned that children who better justified their choices would also be able to properly evaluate their partner’s justification. Therefore, we predicted that justification skills would predict an increased tendency to release the partner in the moral condition.

## Method

### Participants

Participants were 60 3-year-old children (*M* = 3.44, *SD* = 0.28; 32 girls), recruited from a database maintained at the Department of Psychology and from a private nursery, both in the University of Warwick. The sample size was based on previous studies investigating related topics, which made use of similar sample sizes—roughly 24 participants per condition, e.g., [17], with an increased 25% (as we could not predict the effect size). Participants came from families of heterogeneous socioeconomic background, and 3.3% had a racial background other than White (i.e., Black). Additional children were tested but were excluded from the sample because they failed a pre-test task (*N* = 7) or were unable or unwilling to complete the trial (*N* = 8). Prior to the study, parents had given written informed consent for their children to participate in the study.

Children were tested individually in a quiet room in the Psychology Lab or in the Warwick University Nursery between April 2019 and March 2020. Each session lasted approximately 25 minutes. The experiment was conducted in accordance with the Declaration of Helsinki; all procedures were approved by the Humanities & Social Science Research Ethics Sub-committee (HSSREC) at Warwick University.

The study was pre-registered on OSF.org, with the sample size, coding scheme, planned analyses and participant exclusion criteria specified. The pre-registration document is available at https://osf.io/xfr87/.

### Materials and design

In a between-subjects design, each child was assigned to one of the two experimental conditions (selfish motive; moral motive) and received two test trials. Before playing the main game, children received a warm-up, a desire recognition task and a justification task.

**Fig 1:**
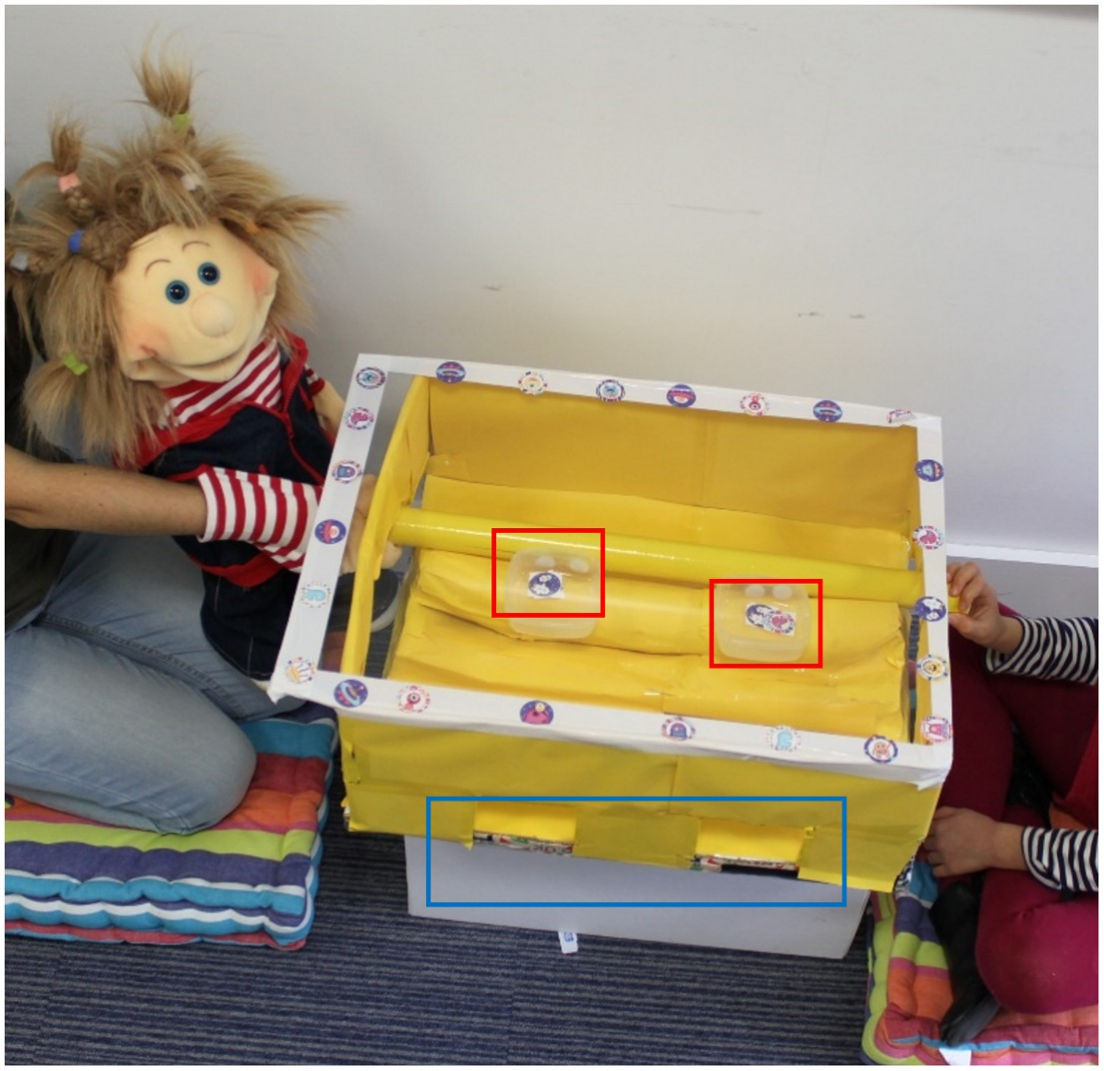
The sticker box game. The child held the tube from their side of the box. When the child and the partner puppet held the tube simultaneously, they were able to push the tube towards the other edge of the box where the two transparent boxes (highlighted in red in the picture) passed through the windows (highlighted in blue in the picture), and they were able to access the stickers.

The materials for the main game included two puppets (the partner puppet and the distracting puppet), sixteen stickers to be collected, two sticker charts, and the ‘sticker box’ (see Fig 1). The sticker box consisted of a cardboard box (50 x 50 x 60 cm), with a transparent lid on top and eight tubes graspable from each side of the box. Five tubes were lined up in the box, while three additional tubes were used to re-bait the box for the second trial.

Additionally, the materials for the warm-up included colored wooden blocks, plastic toy animals and three plastic sheets (featuring three locations). The plastic sheets and additional toy animals were used for the desire recognition task and for the justification task.

### Procedure

#### Warm-up phase

After entering the test room, the experimenter (E) introduced the child to the two puppets (the partner puppet, and the distracting puppet). After a short conversation, the distracting puppet left the room and the partner puppet and E played unrelated warm-up games. Two games served as a pre-test to ensure that children were comfortable talking with the partner puppet: the child had four chances to verbally correct the partner puppet at least once after a mistake (e.g., putting a red block on a blue tower) and thus pass the pre-test.

#### Desire recognition task phase

The desire recognition task consisted in evaluating six toy animals’ mental states considering their previously stated desires. The task was adapted from Rakoczy and colleagues [35] and served to measure participants’ ability to recognize others’ mental states. E placed three plastic sheets (depicting different locations) in front of the child and presented three toy animals which stated their desire to go to their preferred location (all mutually exclusive). Then, E placed all three animals in one fixed location, and asked the child first where each animal wanted to go, and then whether the animal was happy or sad. The task was then repeated once with a second set of animals.

#### Justification task phase

The task consisted in choosing a location for six toy animals and providing a justification to the partner puppet about the choice. The task was adapted from Domberg and colleagues [36] and served to measure participants’ ability to provide justifications for their previous choices. E presented the child with each animal, and the partner puppet asked the child to put the animal in one of the three locations. The partner puppet then asked the child to justify the choice (e.g., *‘Why did you put the polar bear in the sea*?’).

#### Demonstration and training phase

E revealed then the main game (a sticker box) and taught both the child and the partner puppet how to play it. Children could access the stickers placed inside the box by collaborating with the partner puppet. The child and the partner puppet sat by their respective sides of the sticker box, and E instructed them how to get the stickers. If the child and the partner puppet together pushed the upper tube forward toward the other edge of the box, the two little boxes popped out from two little windows and the stickers were free to be collected (see Fig 1). The stickers were placed in two little transparent boxes (one for the child, and one for the puppet) attached to each tube.

The first tube was pushed (and the first two stickers collected) under the supervision of E, who ensured that the child understood the interdependent aspect of the game. E showed the child and the partner puppet a sticker chart, with eight sticker placeholders, that they could use to stick the collected stickers, and left the room.

#### Main game phase (2 trials)

*Joint activity phase*. To create a joint commitment, the partner puppet simply started pushing the tube on their own but failed to move it. If the child did not react, the partner puppet would complain about not getting any sticker. In no circumstance the child and the partner puppet verbally agreed to play together, nor the partner puppet explicitly requested the child to do so. The child and the partner puppet each collected three stickers together (by pushing three of the four remaining tubes). During the game, the partner puppet cheerfully commented on the progress and showed the collected stickers to the child, encouraging the child to do the same. When they have started to push the last tube, the distracting puppet opened the door and stood at the entrance of the room.

*Manipulation phase*. In the selfish motive condition, the distracting puppet interrupted the game and lured the partner puppet to play another game in another room. In the moral motive condition, the distracting puppet interrupted the game by expressing the need for assistance with a small injury.

In both conditions, the distracting puppet stated that she is going to the room next door and then left.

*Test phase*—*open-ended part*. The partner puppet interrupted the sticker game and alternated looking between the child and the door, and after 2 s it started approaching the door. After 2 s the partner puppet addressed the child, repeating the motive for leaving (*‘Alex is in the room next door*! *And /there are many colourful balls there/he needs a puppet to put on a plaster on his finger*!*’*), and then walked towards the door. After 5 more s, the partner puppet again addressed the child asking what to do (*‘What shall I do*?*’*).

*Test phase*—*forced-choice part*. After additional 5 s the partner puppet asked the child whether she should stay in the room or go out to the distracting puppet (*‘Should I stay here*, *or should I go to Alex*?*’*).

Then, the partner puppet left the room, while E entered, re-baited the apparatus and left. The partner puppet entered and played the main game with the child one more time.

*Debrief phase*. After having played the main game phase a second time, E and the child’s caregiver came back to the room and collected with the child all the remaining stickers. E thanked children for playing and gave them a certificate of achievement, the collected stickers, and a small toy.

### Coding and reliability

All the sessions were videotaped. Children’s responses in the desire recognition and justification tasks, as well as children’s reactions to the partner puppet’s failure to finish collecting the stickers were transcribed by a native speaker. Transcripts were then coded by the first author unaware of the condition.

#### Release score

The main measure was whether the child denied or granted the partner puppet release from their joint activity during the test phase. If children explicitly released the partner puppet or manifested signs of release (that is, references to the possibility to follow the distracting puppet to get involved in the alternative activity), they were assigned a score of 0.5. If children instead explicitly protested (i.e., denied release), or manifested signs of protest (i.e., references to the main game), they were assigned a score of -0.5. If children were silent or said something irrelevant, they were assigned a score of 0 (see Table 2 for examples). As each test phase included a part in which children could express freely their mind (open-ended part) and a part in which they were invited to make a choice between two pre-determined choices (forced-choice part), the release score was the results of the sum of the scores of each part. This resulted in a 5-point release score scale (range -1; 1) indicating the level of release that children manifested during the test phase: a score of 1 indicates a consistent expression of release in the two parts of the test phase, whereas a score of -1 indicates a consistent expression of protest.

**Table 2:** Examples of utterances in the open-ended and forced choice parts.

Label	Description	Example	Release Score
Explicit protest	Utterances directed to the partner with reference to main game and involving a normative dimension–with the occurrence of terms such as: must, ought, should, may, right/wrong, good/bad, have to.	‘You should help me take the yellow one out’ ‘You should actually stay here’ ‘We should do this one’	-0.5
	Utterances aimed to re-engage the partner with the main game or indicating that the participant wants the partner to stay and play (including imperatives).	‘Let’s get some more stickers!’ ‘Help me!’ ‘Stay here’	
Signs of protest	Interjections.	‘No!’	
	Utterances directed to the partner aimed to direct the partner’s attention to the fact that the main game is incomplete.	‘There’s one more’ ‘I am not finished yet’ ‘I can’t do this’	
Sign of release	Utterances directed to the partner that refer to the alternative option, including statements aimed to direct the puppet’s attention to the alternative option.	‘Seems he needs some help’ ‘Where is he gone?’ ‘Has he hurt his finger?’	0.5
	Statements that make explicit that the child agrees with the decision of leaving.	‘Oh, OK’	
Explicit release	Utterances directed to the partner that refer to the alternative option and involve a normative dimension (with the occurrence of terms such as: must, ought, should, may, right/wrong, good/bad, have to).	‘You should go to Alex’ ‘You need to go’ ‘You have to give the balls to your friend’	
	Statements aimed to direct the puppet to take up the alternative option, including imperatives.	‘Alex’ ‘Go to Alex’ ‘Help him!’	

#### Desire recognition and justification scores

All children’s responses to the six trials of the desire recognition task were coded as incorrect (0) or correct (1); e.g., if the tiger was placed in the sea and the child responded ‘*the tiger is sad’*, this response was coded as ‘correct’, provided that the trial was valid, i.e., the child correctly recalled the animals’ previously expressed desires (e.g., if the child responded ‘*the tiger wanted to go to the farm*’, thus wrongly recalling the animal’s desire, their response was discarded). Children were assigned an average desire recognition score (0–1) based on the amount of correct answers divided by the total amount of valid trials.

All children’s responses to the partner puppet’s question why children put the animal in the chosen location were scored as relevant (1) or irrelevant (0). Relevant justifications were arguments based on the coding scheme from Domberg and colleagues [36]. Irrelevant justifications included circular arguments (e.g., ‘*because I did it’*), references to the child’s desire (e.g., ‘*because I want so’*) and no responses. Children were assigned an average justification score based on the amount of relevant justifications provided in six trials (0–1).

To establish reliability, a naïve coder blind to the conditions and the hypotheses of the study coded the whole data sample. The two coders were in almost perfect agreement with the Release scores (*Cohen’s k* = 0.96), the desire recognition scores (*Cohen’s k* = 0.91) and the justification scores (*Cohen’s k* = 0.93). Disagreements were solved by discussion between the authors, unaware of the conditions.

## Data analysis

We analyzed children’s reactions by running three separate cumulative link mixed models to assess the effect of the experimental condition on participants’ release scores, and the secondary measures on the release scores from participants who participated in the moral condition. For each subject, we included both trials in the analyses.

The main model included condition, trial number and gender as fixed effects, and participant ID as a random effect. The secondary analysis included models with desire recognition score or justification score, trial number and gender as fixed effects, and participant ID as a random effect. The null models included trial number and gender as fixed effects, and participant ID as a random effect. Model comparison was done using likelihood ratio tests [37, 38]. Statistics were done using R version 3.6.3 [39] and the package ‘ordinal’ [40].

This final analysis presented in the manuscript deviates from the pre-registered version insofar as we decided to build two cumulative link mixed models, which better accommodate ordinal data compared to linear models [41], and following reviewers’ suggestion (See https://osf.io/bhkz8/ for more details).

## Results

In most test trials (84%) children showed a reaction to their partner leaving the main game. Overall, almost one third of the children released (32%) the partner from their joint activity, although they protested (denied release) more often (52%)—and this was true for both conditions. This pattern holds when considering open-ended reactions only: in fact, children spontaneously released the partner (31%), although they were still found to protest more often (52%). With regards to their performances in the secondary tasks, participants presented 63% correct answers in the desire recognition task (*M* = 0.633, *SD* = 0.301) and 50% relevant justification in the justification task (*M* = 0.503, *SD* = 0.408).

### Main model

**Fig 2:**
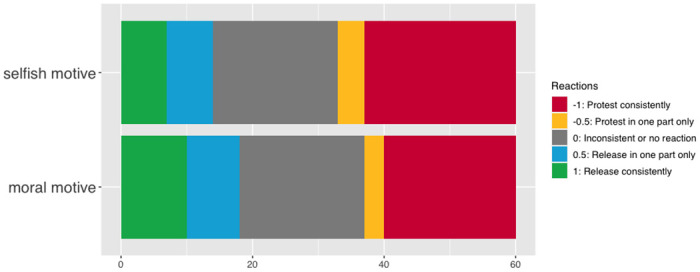
Children’s release scores in the two conditions. Since each participant participated in two test trials, the figure shows up to two scores per participant (one per trial).

We analyzed participants’ release scores in the two conditions. We compared our model with a null model using a likelihood ratio test, but the model did not fit the data better than the null model, *χ*^*2*^(1) = 0.58, *p* = .448 (See https://osf.io/bhkz8/ for more details). There was thus no significant effect of condition on children’s release scores (see Fig 2).

### Secondary analyses

**Fig 3:**
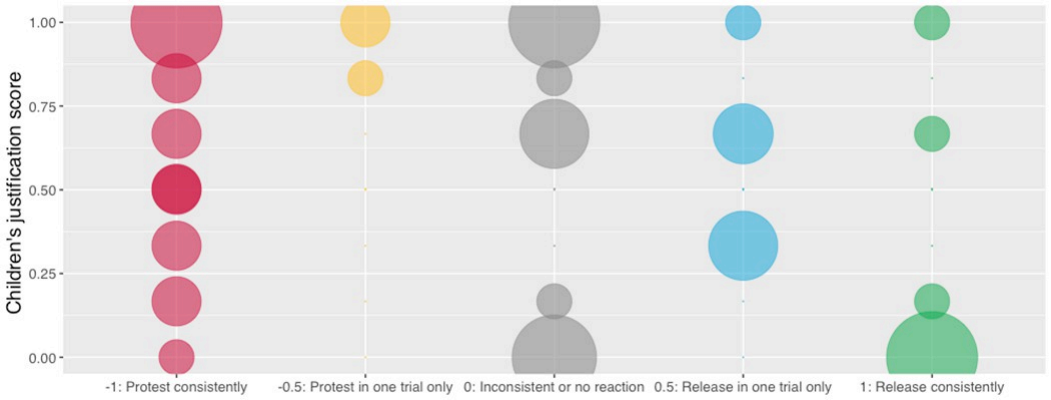
Participants’ distribution of justification scores and release scores in the moral condition. We can see that children with higher justification score were more likely to protest consistently, while children with lower justification score were more likely to consistently release the partner.

We analyzed participants’ release scores in the moral condition in view of their desire recognition scores and their justification scores. We compared our model with a null model using a likelihood ratio test, and the model was a significantly better fit compared to the null model, *χ*^*2*^(1) = 7.57, *p* = .023. The model showed a significant effect of the justification score on the release scores, *estimate* ± *SE* = -2.43, 0.93, *95% CI* [-4.24, -0.61], *χ*^*2*^(1) = 7.52, *p* = .006 (See https://osf.io/bhkz8/ for more details). These results manifest however an unpredicted pattern: a higher justification score predicted a lower release score, indicating children’s tendency (in the moral motive condition) to release the partner puppet less often (see Fig 3). Desire recognition scores and justification scores did not correlate, *t*(58) = -0.170, *p* = .87.

## Discussion

To properly master the concepts of joint activity and collaboration, it is crucial to understand when it is appropriate to interrupt a joint activity. Our results indicate that 3-year-olds did not manifest different reactions when their partner interrupted the joint activity to do something else merely for fun (selfish motive) than to help another agent in distress (moral motive). However, they also show that, when leaving to respond to a moral imperative, children with better argumentative skills tended to release their partners less often.

This is the first study investigating whether participants release their partner from a joint activity (and hence from the implicit commitment arising from it) when the joint activity conflicts with a moral duty. In this study participants and their partner engaged in a repeated joint activity in which their outcomes were interdependent, so even though commitments cannot be measured directly, it is plausible to assume that a certain level of implicit commitment between participants and their partner was reached. We believe that the fact that some children used normative language both when they released (18%) and when they protested (19%) in the open-ended part supports this assumption about implicit commitment emerging from repeated joint action and interdependence of outcomes. The spontaneous use of normative language is particularly striking given that our paradigm did not involve any verbal commitment or agreement to play, and the experimenters never used normative or we-language, in contrast to most studies in the literature [with some exceptions; see e.g., 19]. This finding is consistent with the idea that commitment should be considered a ‘graded’ phenomenon, and not a binary one, and as such does not necessarily need to be verbally explicit to have some normative power [3, 16].

While 3-year-old children have been found to distinguish between intentional and unintentional commitment violations [17], and between excused and unexcused commitment violations [42], our results did not show that 3-year-olds evaluate the scope of implicit commitment. This is consistent with recent findings showing that young children are reluctant to break their own promises, irrespective of the motive to break them [18]—but contrary to [8], who found that 3-year-olds judge defection less harshly when there is a good justification (although the justification did not impact other judgements such as tattling, liking, or partner choices). Li and colleagues manipulated their moral (prosocial) and selfish motives in a similar way to our own manipulation (i.e., helping someone vs. engaging in a more fun activity); on the other hand, the action that the partner was required to do to fulfil their commitment (showing a toy) could be perceived as not inconsistent with the justification provided as selfish justification (bad excuse). Therefore, the selfish justification was not only selfish, but potentially irrelevant. Our own contradicting finding, instead, could be due to the fact that our selfish motive condition may still be interpreted as a relevant motive, albeit selfish, and therefore taming our manipulation.

This is also the first study measuring participants’ tendency *to release* their partner from a previous unfinished joint activity. In present study, we showed that 3-year-olds can take initiatives to release their partners, although less frequently as they take initiative to protest. On the other hand, this is striking considering that releasing requires a more complex assessment of the situation.

Our secondary aim was to investigate to what extent children’s understanding of the scope of joint action is related to their understanding of others’ mental states and justificatory abilities. We reasoned that children who better justified their choices would also be able to properly evaluate their partner’s justification. This prediction would be supported by Vygotskian theories of reasoning [43–45], according to which humans developed reasoning skills in order to reach cooperative decisions, as well as argumentative theory of reasoning [31, see also 46], according to which the primary function of reason is to win arguments with social partners. Furthermore, by the age of three, children have been found able to collaboratively reason and make references to shared values with their social partners [47], suggesting that in our setting children would take into account the partner’s motive to help a third agent in distress. Surprisingly, our data showed that children who were better able to justify their own choice (i.e., had higher justification scores) were instead less inclined to release their partner in the moral condition. We may speculate that the children who were more adept at providing reasons to support arguments were more accustomed to getting what they wanted, and that their argumentative skills may subserve self-interest, be it mutual or individual. This finding can suggest that the (joint) goal established during the playful activity was binding enough to overcome other considerations, and that children still overestimate the scope of commitment, assuming the existence of commitments when they are not in place or not recognizing when they should be dissolved [48].

Our study raises additional new questions for further research. Do participants manifest additional implicit expectations about the scope of joint actions and, possibly, commitments? Would these expectations differ across multiple cultures (or sub-cultures), in particular across non-WEIRD participants [49, 50]? Also, given that protests can be considered strategies of partner control [16], it would be interesting to assess whether a partner’s tendency to release has an impact on participants’ partner choice strategies, i.e., whether participants prefer to interact more with partners who are more likely to release them in certain situations [51, 52, see also 53]. Furthermore, it would be interesting to compare conditions in which the level of coordination and repetition are modulated, and to measure how this impacts the release and protest rates; in line with [23, 54], we expect that coordinated activities lead to the emergence of a sense of commitment, and therefore the lack of it would lead to a drop in children’s protests.

In summary, although children actively responded when their partner interrupted the joint activity to do something else, their reactions did not differ according to partner’s motive. However, children demonstrated that they are able to actively release their partner from their previous unfinished joint activity, even though it meant that they would not complete the activity itself. This indicates that measuring release—in addition to measuring protest—is a new useful tool for studying joint actions, collaboration, and commitment.
